# Validation on the First-Tier Fully Automated High-Throughput *SMN1*, *SMN2*, TREC, and *RPP30* Quantification by Quadruplex Droplet Digital PCR for Newborn Screening for Spinal Muscular Atrophy and Severe Combined Immunodeficiency

**DOI:** 10.3390/ijns11040097

**Published:** 2025-10-19

**Authors:** Chloe Miu Mak, Timothy Yiu Cheong Ho, Man Kwan Yip, Felicite Enyu Song, Raymond Chiu Mo Tam, Leanne Wing Ying Yu, Ann Anhong Ke, Eric Chun Yiu Law, Toby Chun Hei Chan, Matthew Chun Wing Yeung

**Affiliations:** Newborn Screening Laboratory, Department of Pathology, Hong Kong Children’s Hospital, Hong Kong SAR, China; timothy.ho@ha.org.hk (T.Y.C.H.); ymk042@ha.org.hk (M.K.Y.); fe.song@ha.org.hk (F.E.S.); tcm203@ha.org.hk (R.C.M.T.); leanne.yu@ha.org.hk (L.W.Y.Y.); ann.ke@ha.org.hk (A.A.K.); h1094168@connect.hku.hk (E.C.Y.L.); cch191@ha.org.hk (T.C.H.C.); ycw186@ha.org.hk (M.C.W.Y.)

**Keywords:** digital PCR, dried blood spots, multiplex, newborn screening, spinal muscular atrophy, *SMN1*, *SMN2*, severe combined immunodeficiency, T-cell receptor excision circle (TREC)

## Abstract

Newborn screening (NBS) for spinal muscular atrophy (SMA) and severe combined immunodeficiency (SCID) faces challenges. Accurate and precise *SMN1* and *SMN2* copy number determination, confirmed by two orthogonal methods, are vital for SMA prognostication and treatment. Single *SMN1* copy detection also enables the further feasibility to screen for compound heterozygotes. In SCID, low-level T-cell receptor excision circle (TREC) quantification by quantitative PCR is imprecise, necessitating replicates for reliable results. An assay with enhanced accuracy, precision, and high throughput is warranted for NBS SMA and SCID. False positive of *SMN1* deletions due to allele dropout are also a potential pitfall in PCR-based methods. We evaluated a first-tier fully automated quadruplex droplet digital PCR (ddPCR) assay detecting *SMN1*, *SMN2*, TREC, and *RPP30* using dried blood spots together with a second-tier Sanger sequencing to exclude *SMN1* allele dropout. Five proficiency test samples and six patient samples with known *SMN1* and *SMN2* copy numbers confirmed by multiplex ligation-dependent probe amplification were used for accuracy evaluation with full concordance. The ddPCR assay showed high precision for *SMN1* and *SMN2* (<7% coefficient of variation (CV) for ≥0 copy) and TREC (14.6% CV at 37 copies/µL blood). Second-tier Sanger sequencing identified all SMA cases with homozygous deletions. Accuracy for TREC classification was concordant with 10 proficiency samples. The reference interval of TREC concentration was established for newborns ≥ 34 weeks (*n* = 1812) and the 2.5th percentile was 57 copies/µL blood. A two-tiered approach with fully automated quadruplex ddPCR and Sanger sequencing delivers accurate and precise quantitation for NBS SMA and SCID, enabling early treatment and counseling.

## 1. Introduction

Spinal muscular atrophy (SMA) and severe combined immunodeficiency (SCID) are genetic disorders that demand timely newborn screening (NBS) due to their rapid and severe progression. The first-tier NBS of both diseases involves the detection of molecular markers, viz. *SMN1* and T-cell receptor excision circle (TREC), which are often multiplexed [1,2,3].

SMA is an autosomal recessive neurodegenerative disease predominantly arising from homozygous deletions of exon 7 within the *SMN1* gene, precipitating a deficiency in the SMN protein [4]. Epidemiologically, the prevalence of SMA varies across NBS programs globally, with reported rates of 1 in 13,309, 1 in 27,962, and 1 in 11,471 in the United States, Canada, and China, respectively [5,6]. SMA presents a spectrum of severity across five distinct phenotypes, each characterized by different ages of onset (types 0 through 4). Type 1, for instance, exhibits a 90% degeneration of motor neurons by six months of age [7]. This underscores the need for prompt diagnosis and therapeutic intervention by modalities such as antisense oligonucleotides or gene therapy [7].

The clinical manifestation of SMA is modulated by the copy number of the *SMN2* gene, a paralog of *SMN1* that differs by only five nucleotides. *SMN2* can partially compensate for *SMN1* loss, producing approximately 10% of full-length SMN protein [8]. As a result, the *SMN2* copy number exhibits an inverse correlation with phenotypic severity and provides critical prognostic insights [9]. Individuals with SMA typically have a *SMN2* copy number variation between one and six [10]. The copy number variation in SMN2 plays a game-changing role in determining the need for treatment. The treatment approach for four copies of *SMN2* has been an ongoing debate. Experts in China and the United States recommend early treatment, while Germany recommends a follow up strategy of “watchful waiting” [11,12,13].

Contemporary NBS methodologies encompass quantitative PCR (qPCR), DNA melting curve analysis, digital PCR (dPCR), and potentially next-generation sequencing (NGS) to detect homozygous *SMN1* exon 7 deletions. The qPCR remains the most widely used first-tier screening method despite its large imprecision, while multiplex ligation-dependent probe amplification (MLPA) and dPCR are often applied as diagnostic methods for confirming NBS results [3,5,14,15,16]. MLPA typically has a longer assay time and relatively high cost [17], whereas NGS would require specialized bioinformatics tools for result interpretation, long-read sequencing, or prior long-range PCR due to the high homology between *SMN1* and *SMN2* [15]. Detection of a single *SMN1* copy can help identify 2–5% of individuals with compound heterozygosity who require supplementary long-read sequencing or long-range PCR prior to NGS to uncover other *SMN1* small mutations [10]. Timely evaluation of *SMN2* copy number is indispensable for therapeutic strategizing; however, it is not performed by every screening laboratory for positive cases [3]. Discrepancies may also arise across or within diagnostic platforms; significant variations in *SMN2* copy numbers were reported within the batches of MLPA analysis [18,19,20]. This prompts experts to endorse dual testing by two independent laboratories by two different and independent methods advocating for standardization of laboratory procedure in determining *SMN2* copy number [10,21]. Early evaluation also allows for timely intervention, as newborns treated before six weeks of age show improved development compared to those treated later. SMA is considered as a time-critical condition (https://www.ncbi.nlm.nih.gov/books/NBK55827/ (accessed on 25 September 2025)). Therefore, the ideal timeframe for concurrent *SMN1* and *SMN2* copy number analyses, which require expertise in genetics, should be available as soon as possible to enable clinicians to deliver early interventions within 3–6 weeks [10]. On the other hand, false positives due to allele dropout in genotyping assays have been reported for *SMN1* hybrids or probe-binding site variants [22]. Therefore, it is prudent to confirm the first-tier SMA-positive results with a second-tier orthogonal method using probes targeting another nucleotide differentiating *SMN1* and *SMN2* before labeling such a potentially lethal disease that necessitates urgent and ultra-expensive treatment [10].

SCID is a group of inherited disorders characterized by profound T- and B-cell deficiencies, with an incidence of approximately 1 in 58,000 births [23]. Early detection of SCID through NBS is crucial, as untreated SCID often leads to early mortality due to severe infections. However, hematopoietic stem cell transplantation offers survival rates exceeding 80% for newborns treated shortly after birth. NBS relies on measuring low-level TREC in dried blood spots (DBS) to identify T-cell deficiencies. While qPCR with DBS is widely used in NBS for SMA and SCID due to its simplicity, short assay time, and lower cost, it requires a standard curve for calibration and typically needs replicate testing to improve precision at low TREC levels. In contrast, dPCR offers a superior alternative due to its exceptional sensitivity to low-level targets, precision, and absolute quantification without the need for standard curves and resilience against PCR inhibitors, making it well-suited for DBS analysis [24,25]. Studies have demonstrated the commendable performance of dPCR in quantifying *SMN1*, *SMN2*, and TREC individually [24,25,26,27]. However, to our knowledge, a single fully automated quadruplex assay combining *SMN1*, *SMN2*, TREC, and the reference gene *RPP30* has yet to be evaluated in a newborn screening laboratory. Such an assay could simultaneously detect *SMN1* deletions for SMA, utilize *SMN2* copy number for just-in-time prognostication, and identify SCID through low-level TREC in a streamlined run, optimizing early intervention for these life-threatening conditions

To address these technical challenges, we evaluated a first-tier quadruplex droplet dPCR (ddPCR) assay for NBS SMA and SCID simultaneously detecting *SMN1*, *SMN2*, TREC, and *RPP30*, paired with a second-tier Sanger sequencing for *SMN1*.

## 2. Materials and Methods

### 2.1. Specimens

The study was conducted in accordance with the Declaration of Helsinki and approved by the Central Institutional Review Board of Hong Kong Hospital Authority (PAED-2023-088) on 5 February 2024. Archived DBS from newborns aged 24 h to 72 h using Whatman 903^TM^ Protein Saver Card (DBS card) (Cytiva, Washington, DC, USA) were retrieved for reference interval establishment. The cards were stored at -20 °C with desiccant in sealed plastic bags [28]. In-house prepared samples and samples from the Centers for Disease Control and Prevention Newborn Screening Quality Assurance Program (CDC NSQAP) were used for precision and accuracy analysis. A total of 1867 samples were analyzed to study the distribution of *SMN1* and *SMN2* copy numbers. Only full-term samples (≥34 weeks gestation, *n* = 1812) were used to establish the TREC reference interval. At the time of study, there were only 55 pre-term samples, which were not adequate for reference interval establishment.

### 2.2. First-Tier Quadruplex ddPCR of SMN1, SMN2, TREC, and RPP30

#### 2.2.1. DNA Extraction from DBS

A single 3.2 mm disk was punched from DBS using the Panthera Puncher^TM^ 9 (Revvity, MA, USA) into a full-skirted 96-well plate (Eppendorf, Hamburg, Germany). DNA extraction was performed with QuantaBio Extracta^TM^ DBS solution (QuantaBio, MA, USA). In brief, the disks were washed with 100 µL DBS reagent, followed by a 5 min centrifugation at 2200× *g*. After removing the supernatant, 50 µL DBS reagent was added to each well. The plate was then centrifuged for 30 s at 2200× *g* and shaken at 95 °C, 700 rpm for 20 min, followed by cooling at 4 °C for 1 min. Finally, the plate was centrifuged at 3500× *g* for 10 min to sediment the debris.

#### 2.2.2. ddPCR Method

The fully automated ddPCR multiplexing *SMN1*, *SMN2*, TREC, and *RPP30* quadruplex assay (Cat no: 12003909, Unique Assay ID: dHsaEXD54223818, Bio-Rad, Pleasanton, CA, USA) [29] was evaluated. *SMN1*, *RPP30*, *SMN2*, and TREC were assigned to FAM, HEX, Cy5, and Cy5.5 channels, respectively. A 24 µL ddPCR reaction contained 6 µL ddPCR of multiplex supermix (Cat no: 12005910, Bio-Rad), 1 µL quadruplex assay, 0.3 µL 300 mM dithiothreitol (Cat no: 12012171, Bio-Rad), 7 µL DNAse free water, and 10 µL of DBS DNA sample. Then, 20 µL of each reaction was transferred to the GCR96 cartridge (Cat no: 12006859, Bio-Rad) by a multichannel pipette. The cartridge was sealed twice using the PX1 plate sealer (Bio-Rad) at 180 °C for 0.5 s each time. The GCR96 cartridge was centrifuged at 1150× *g* for 30 s. Finally, the cartridge was loaded onto the QX ONE ddPCR System (Bio-Rad). The plate template from the puncher was uploaded to the ddPCR system software 1.3 Standard Edition. The thermal cycling protocol consisted of 25 °C for 1 min, 95 °C for 10 min, followed by 40 cycles of denaturation at 94 °C for 30 s, annealing and extension at 58 °C for 1 min, and enzyme deactivation at 98 °C for 10 min with ramp rate of 2 °C per second. Once initiated, the GCR96 cartridge was automatically processed in the QX ONE system, allowing for multiple cartridges to be loaded and processed sequentially.

The QX ONE software version 1.3 Standard Edition (Bio-Rad) for the QX ONE ddPCR system was used for ddPCR plot analysis. Wells with an accepted event count of <10,000 were excluded [30]. The fluorescent threshold was applied to the region with the lowest droplet density between the clusters. *RPP30* was set as the reference with a copy number of two. The *SMN1* and *SMN2* copy numbers were calculated automatically by the software. The *RPP30* and TREC concentrations were converted from copies/µL per 20 μL well in the software to copies/µL blood. The conversion was performed as the following: one 3.2 mm disk contains 3 μL of blood, DNA was eluted in 15 μL of water, and 8 μL of DNA was used in the final PCR; copies/μL blood = (copies per 20 μL well/8 μL input DNA) × (15 μL elution/3 μL blood per punch) [24]. The recommended *RPP30* loading in the reaction should be 100 to 5000 copies/µL according to the manufacturer, which corresponds to 4167 to 208,350 copies/µL blood after conversion. Therefore, a minimum *RPP30* input of 4200 copies/µL blood was considered as a cutoff to ensure sufficient DNA was loaded for *SMN1* and *SMN2* copy number determination and TREC quantification.

#### 2.2.3. Precision

We conducted 20 replicate measurements on the same day to evaluate within-run precision and performed 25 replicate measurements across five separate days to determine between-run precision with the in-house prepared samples. This analysis included four samples with 0–3 copy numbers of *SMN1* exon 7, five samples with 0 – 4 copy numbers of *SMN2* exon 7, and two samples with TREC concentrations at low and high levels. In subsequent internal quality control practice, the precision performance was monitored using Levey–Jennings charts, with acceptance criteria set at ±2 standard deviations (SD) from the mean, following Westgard rules.

#### 2.2.4. Accuracy

Five DBS samples from CDC NSQAP *SMN1* Exon 7 Analysis external quality assurance program and six SMA patient samples (with 0 *SMN1* and 2–4 *SMN2* copies, confirmed with MLPA) as well as ten DBS samples from the CDC’s NSQAP TREC in Dried Blood Spots Proficiency Testing Program (TRECPT) and two SCID patient samples were used for accuracy study.

#### 2.2.5. Limit of Blank, Limit of Detection, and Limit of Quantification

The limit of blank (LoB) was calculated from 20 replicate measurements of blank DBS card punches. The limit of detection (LoD) was determined using the formula LoD = LoB + 1.6 × SD, based on 20 replicates of an in-house prepared DBS sample. The limit of quantification (LoQ) is generally defined as the lowest concentration at which an analyte can be reliably quantified, with a coefficient of variation (CV) of <25% used for dPCR. The LoQ of *SMN1*, *SMN2*, *RPP30*, and TREC were determined using 20 replicate measurements from an in-house prepared DBS sample.

#### 2.2.6. Linearity of TREC Measurement

Synthetic TREC oligonucleotide was introduced to a TREC-free whole blood matrix. A two-fold serial dilution was performed, generating eight concentration levels from 20 to 2500 TREC copies/µL blood, each measured in five replicates. The measured TREC concentrations were fitted to a linear regression analysis.

#### 2.2.7. Storage Stability

DNA fragmentation increases with extended storage duration; thus, the storage stability of DBS specimens was evaluated [31]. The stability of 28 specimens stored at -20 °C and subjected to six freeze-thaw cycles, as well as 10 specimens maintained at ambient temperature, were assessed for a 14-day period, respectively. The mean recoveries of each target (*SMN1*, *SMN2*, *RPP30*, and TREC) on day 14 were compared to day 0.

### 2.3. Second-Tier Sanger Sequencing for SMN1 and SMN2 Exon 7

The MANE SELECT transcripts used for *SMN1* and *SMN2* were NM_000344.4 and NM_017411.4, respectively. Since the primers of first-tier ddPCR screening targeted its 3′ end on c.840C for *SMN1* and c.840T for *SMN2*, the second-tier test aimed to avoid false positives due to allele dropout caused by other nucleotide variations at c.840 such as c.840C>G (rs1164325688 with allele frequency 0.000001240 gnomAD v4.1.0).

Assay A was *SMN1*-specific. The 3’ end primer sites targeted two other nucleotides differentiating *SMN1* from *SMN2*, which are c.835-44G>A on forward primer and c.888+100A>G on reverse primer, respectively. Assay B was non-specific and would amplify both *SMN1* and *SMN2*. In the case of homozygous *SMN1* exon 7 deletion detected by first-tier ddPCR, assay A would show no amplicon and assay B would show amplicon signal of homozygous c.840T by amplifying *SMN2*. In the case of false positives of *SMN1* exon 7 deletion due to allele dropout at c.840, for example, homozygous c.840G, assay A would show homozygous c.840G and assay B would show amplicon signals of heterozygous c.840G/T.

Primer sequences of assay A were published before and tagged with M13 (indicated in bold): assay-A-F: 5′-TGTAAAACGACGGCCAGT-CTA ATT TTT TGT ACT TTC AGT AGA AAT and assay-A-R: 5′-CAGGAAACAGCTATGACC-ACA TTA ACC TTT CAA CTT TT [32]. PCR conditions were conducted at 95 °C for 10 min followed by 40 cycles of 95 °C for 30 s, 55 °C for 30 s, and 72 °C for 60 s, and a final elongation of 72 °C for 7 min.

Primer sequences of assay-B-F were TGTAAAACGACGGCCAGT-AAC CTT AAC TGC AGC CTA ATA ATT G and assay-B-R CAGGAAACAGCTATGACC-GCT GGC AGA CTT ACT CCT TAA T [33]. The PCR conditions were conducted at 95 °C for 10 min followed by 40 cycles of 95 °C for 30 s, 62 °C for 30 s, and 72 °C for 60 s, and a final elongation of 72 °C for 7 min.

Six 3 mm disks were punched from each DBS specimen in a 1.5 mL tube. DNA was extracted using QIAamp DNA Mini Kit (QIAGEN, Venlo, The Netherlands). Amplicons were analyzed by Agilent 2100 Bioanalyzer System (Agilent, Santa Clara, CA, USA) and sequenced on a 3500xL Genetic Analyzer. All PCR reagents and sequencing reagents were purchased from Thermo Fisher Scientific (Waltham, MA, USA).

### 2.4. Statistical Analysis

The Analyze-it Ultimate Edition software version 6.15 (Analyze-it, Leeds, UK) for Microsoft Excel (Microsoft, Redmond, WA, USA) was used for data management and statistical analysis. Assay precision was analyzed and plotted with Prism 5.0 (GraphPad Software, Boston, MA, USA).

## 3. Results

**Figure 1:**
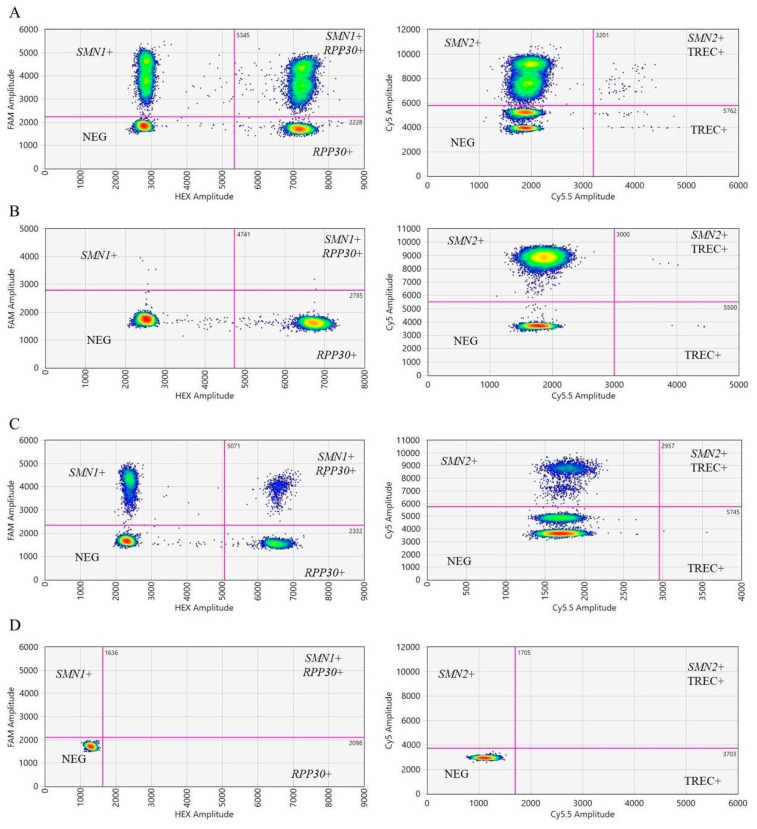
The 2D heatmap plots of ddPCR droplet clusters generated by QX ONE Software. Channels 1, 2, 3, and 4 detect *SMN1*, *RPP30*, *SMN2*, and TREC, respectively. (**A**) Normal control; (**B**) positive SMA control with homozygous *SMN1* exon 7 deletion showed absence of *SMN1* cluster; (**C**) positive SCID control without TREC cluster; (**D**) no-template control.

Droplets were clustered in 2D plots for quantification using QX ONE software in Figure 1. The assays applied to the QX ONE system in each reaction were represented by two 2D plots with each axis representing one channel.

### 3.1. Precision

**Figure 2:**
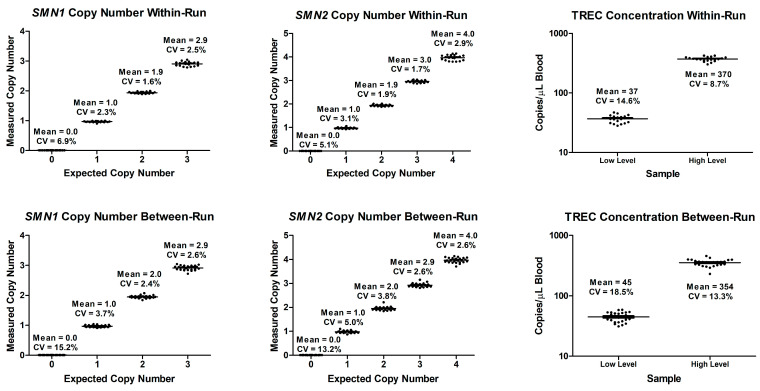
Within-run and between-run precisions of *SMN1*, *SMN2*, and TREC assays.

In within-run CV for *SMN1* and *SMN2* at 0 copies were 6.9% and 5.1%, respectively, and both at ≥1 copy was ≤3.1%. In between-run CV for *SMN1* and *SMN2* at 0 copies were 15.2% and 13.2%, respectively, and both at ≥1 copy was ≤5.0%. For TREC, the within-run CV at 37 and 370 TREC copies/µL blood were 14.6% and 8.7%, respectively. The between-run CV at 45 and 354 TREC copies/µL blood were 18.5% and 13.3%, respectively. Figure 2 shows the within-run and between-run precision.

### 3.2. Accuracy

All samples from the CDC NSQAP and SMA patients demonstrated 100% concordance on both ddPCR and Sanger sequencing results (Table 1). All CDC NSQAP TRECPT samples for TREC measurement also exhibited 100% concordance (Table 2). In addition, two SCID patient samples showed 0 TREC copies/µL blood.

### 3.3. Limit of Blank, Limit of Detection, and Limit of Quantification

LoB for *SMN1*, *SMN2*, *RPP30*, and TREC were 0 copies/µL blood. LoD for *SMN1* and *SMN2* were 1.0 copy with CV of 2.3% and 3.1%, respectively. For TREC and *RPP30*, LoD were 3 copies/µL blood with CV of 32.7% and 80 copies/µL blood with CV of 5.5%, respectively. LoQ were 1.0 copy for *SMN1* and *SMN2*, 29 copies/µL blood for TREC and 904 copies/µL blood for RPP30.

### 3.4. Linearity of TREC Measurement

The two-fold serial dilution results for TREC were analyzed, achieving an *R*^2^ value > 0.99, indicating satisfied linearity.

### 3.5. Storage Stability

Mean recoveries for all targets (*SMN1*, *SMN2*, TREC, and *RPP30*) were within ±10%, under both ambient temperature and -20 °C over 14 days storage.

### 3.6. Distribution Study of SMN1 and SMN2 Copy Numbers

Among the normal samples, the *SMN1* copy number ranged from 1 to 5 with one copy accounting for 2.3%, while *SMN2* copy number ranged from 0 to 6 with four copies accounting for 0.3% (Table 3).

### 3.7. Reference Interval of TREC Concentration

The 2.5th percentile was 57 copies/µL blood, which was used as a screening cutoff for re-punch, retest, and recall in newborns with a gestational age of ≥34 weeks.

## 4. Discussion

In Hong Kong, a territory-wide NBS program for glucose-6-phosphate dehydrogenase deficiency and congenital hypothyroidism was started in 1984 [34]. In 2015, the program expanded to include NBS of inborn errors of metabolism using DBS in the public sector, which further extended to NBS for SCID in 2021 and NBS for SMA in 2023.

**Figure 3:**
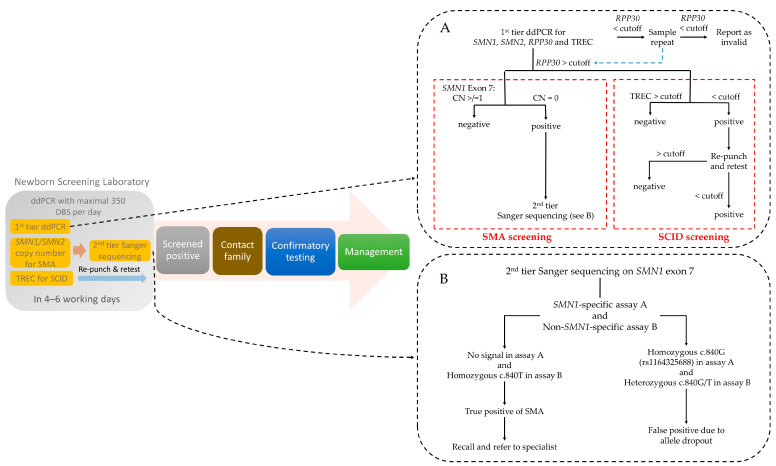
Laboratory workflow of NBS for SMA and SCID. (**A**) First-tier screening of *SMN1*, *SMN2*, TREC, and *RPP30* with ddPCR method. CN, copy number; (**B**) Second-tier screening of *SMN1* exon 7 with Sanger sequencing.

Here, we successfully validated the first-tier, quadruplex, high-throughput, and fully automated ddPCR method on NBS for both SCID and SMA (Figure 3A). The quadruplex reaction for simultaneous detection of *SMN1*, *SMN2*, TREC, and *RPP30* in a single reaction enables a short turnaround time to minimize the treatment latency of SMA and SCID patients. The ddPCR demonstrated good precision in measuring the copy number of *SMN1* and *SMN2* in a 3.2 mm DBS spot. For ≥1 copy number of *SMN1* and *SMN2,* the between-run CV was ≤5%. There was no mid-integer observed at any copy number level for both *SMN1* and *SMN2*. The timely and accurate information on *SMN2* copy number aids medical professionals in making felicitous clinical decisions and providing specific information regarding the prognostication and treatment plan in genetic counseling [2,8].

Currently, most if not all NBS programs for SMA only aim to detect homozygous exon 7 deletion in *SMN1*. However, there are still 2 – 5% of SMA patients harboring heterozygous exon 7 deletion and another small mutation in *SMN1*, which would be missed in this approach. The application of ddPCR aids in detecting newborns with one copy of *SMN1*, which can provide a possibility for further genetic tests to screen for compound heterozygous mutations, such as long-read sequencing or long-range PCR prior to NGS [35,36].

In our study, ddPCR also demonstrated good precision in measuring the low TREC concentrations reducing the need for re-punch and retest. Since there is no standardization in the method, each laboratory should establish their own reference intervals and cutoff. The numerical values are not interchangeable and can be affected by differences in DNA extraction methods and dPCR assay designs. Moreover, dPCR offers a key advantage over qPCR and DNA melting curve analysis by enabling absolute quantification without requiring a calibration curve. Automated dPCR platforms can meet the high throughput requirement in NBS programs, processing over 350 samples daily per instrument with a comparable reagent price of approximately USD 15 – 20 per test in a quadruplex assay.

We also employed a second-tier Sanger sequencing to mitigate the risk of false positives due to allele dropout (Figure 3B). Although the allele frequency of other nucleotide changes at c.840 is low, the implication of a screen-positive SMA result for this time-critical condition is serious and hence it is prudent to perform a second-tier test prior to patient recall. In differentiating the true gene *SMN1* from its pseudogene *SMN2*, only a few SNPs along the gene region are available. False positives due to allele dropout at such SNPs have been reported [22]. More importantly, the probes for *SMN1* exon 7 in PCR-based methods, such as MLPA, qPCR, and dPCR, are usually targeted on c.840C>T. In order to avoid the risk of false positives due to allele dropout, we employed a second-tier test by Sanger sequencing targeted on two other SNPs, namely c.835-44G>A and c.888+100A>G.

Regarding the limitations, dPCR is more technically demanding in experiment design and data analysis as well as a higher capital cost compared to qPCR and DNA melting curve analysis. Moreover, our present assay design cannot detect partial intragenic *SMN2* deletions, hybrid gene structures, single nucleotide variants, and other modifier genes. As the availability of dPCR platforms continues to grow, increased competition may lead to lower prices, making its use in routine medical laboratories more widespread.

## 5. Conclusions

We successfully evaluated a first-tier, quadruplex, high-throughput, and fully automated ddPCR assay for *SMN1*, *SMN2*, TREC, and *RPP30* and second-tier *SMN1* Sanger sequencing for NBS SMA and SCID. The method enables high throughput screening capacity, while precisely quantifying the *SMN2* gene with a fast turnaround time and reducing the risk of false positives in *SMN1* due to allele dropout. The method is already in use for the NBS for SMA and SCID in Hong Kong since October 2023.

## Figures and Tables

**Table 1:** Accuracy comparison with CDC NSQAP samples and patient samples for *SMN1* and *SMN2* measurements.

**NSQAP Specimens**	**NSQAP Classification**		**Copy Number Results by** **First-Tier ddPCR**		**Our Classification**		
			***SMN1***	***SMN2***			
1	Normal		2.0	1.0	Normal		
2	SMA		0.0	1.9	SMA		
3	Carrier		0.9	1.9	Carrier		
4	SMA		0.0	1.9	SMA		
5	Carrier		0.9	1.9	Carrier		
**SMA Patient Specimens**	**Copy Number Results by MLPA**		**Copy Number Results by** **First-Tier ddPCR**		**Second-Tier Sanger Sequencing** **Results**		
	***SMN1***	***SMN2***	***SMN1***	***SMN2***	**Assay A**	**Assay B**	***SMN1* Exon 7**
1	0	2	0.0	2.0	No peak	c.840T	Absent
2	0	2	0.0	2.0	No peak	c.840T	Absent
3	0	3	0.0	3.0	No peak	c.840T	Absent
4	0	3	0.0	3.1	No peak	c.840T	Absent
5	0	4	0.0	4.0	No peak	c.840T	Absent
6	0	4	0.0	4.0	No peak	c.840T	Absent

**Table 2:** Accuracy comparison with CDC NSQAP TRECPT samples for TREC measurements.

Specimen No.	ddPCR Results			NSQAP Results
	TREC Copies/µL Blood ^1^	*RPP30* Copies/µL Blood ^2^	Our Classification	
1	326	10,932	Negative	Negative
2	130	14,472	Negative	Negative
3	141	15,927	Negative	Negative
4	168	9998	Negative	Negative
5	0	144,892	Positive	Positive
6	0	122,741	Positive	Positive
7	0	120,351	Positive	Positive
8	0	73,699	Positive	Positive
9	0	0	Unqualified sample	Unqualified sample
10	0	4	Unqualified sample	Unqualified sample

^1^ TREC of >57 copies/µL blood considered as normal. ^2^ *RPP30* of >4200 copies/µL blood considered as qualified sample.

**Table 3:** Distribution of *SMN1* and *SMN2* copy number in NBS samples (*n* = 1867).

Gene	Copy Number	Sample Number	Frequency, %
*SMN1*	1	43	2.3
	2	1707	91.4
	3	111	6.0
	4	4	0.2
	5	2	0.1
*SMN2*	0	90	4.8
	1	669	35.7
	2	1040	55.7
	3	57	3.1
	4	5	0.3
	5	5	0.3
	6	1	0.1

## Data Availability

The data presented in this study are available on request from the corresponding author.

## Abbreviations

The following abbreviations are used in this manuscript:

CDC	Centers for Disease Control and Prevention
CV	Coefficient of variation
DBS	Dried blood spots
ddPCR	Droplet digital PCR
LoB	Limit of blank
LoD	Limit of detection
LoQ	Limit of quantification
MLPA	Multiplex ligation-dependent probe amplification
NBS	Newborn screening
NGS	Next-generation sequencing
NSQAP	Newborn screening quality assurance program
qPCR	Quantitative PCR
SCID	Severe combined immunodeficiency
SD	Standard deviation
SMA	Spinal muscular atrophy
TREC	T-cell receptor excision circle
